# Generation of human parallel chimeric antigen receptor (pCAR) T cells to achieve synergistic T cell co-stimulation

**DOI:** 10.1016/j.xpro.2022.101414

**Published:** 2022-05-21

**Authors:** Daniel Larcombe-Young, Lynsey Whilding, David Marc Davies, Benjamin Draper, Natasha Bechman, John Maher

**Affiliations:** 1 School of Cancer and Pharmaceutical Sciences, King’s College London, Faculty of Life Sciences and Medicine, Guy’s Campus, London SE1 9RT, UK; 2 Leucid Bio Ltd., Guy’s Hospital, Great Maze Pond, London SE1 9RT, UK; 3 Department of Immunology, Eastbourne Hospital, Kings Drive, Eastbourne, East Sussex BN21 2UD, UK

**Keywords:** Cell Biology, Cell isolation, Flow Cytometry/Mass Cytometry, Cell-based Assays, Cancer, Immunology, Molecular Biology

## Abstract

Dual co-stimulation may be harnessed using parallel chimeric antigen receptors (pCARs) in which two distinct co-stimulatory units are adjacently localized on the plasma membrane. This protocol summarizes construct design, human T cell isolation, retroviral transduction, tissue culture expansion, and preclinical testing of pCAR T cells, exemplified by receptors that co-target avb6 integrin and ErbB dimers.

For complete details on the use and execution of this protocol, please refer to Muliaditan et al. (2021).

## Highlights

• A protocol to enable robust generation of pCAR T cells
• Effective dual co-stimulation is provided by this platform
• Sustained and enhanced antitumor activity by pCAR T cells
• Detailed *in vitro* and *in vivo* evaluation of pCAR technology

## Abstract (editor highlights)

Publisher’s note: Undertaking any experimental protocol requires adherence to local institutional guidelines for laboratory safety and ethics.

## Graphical abstract

**Figure:**
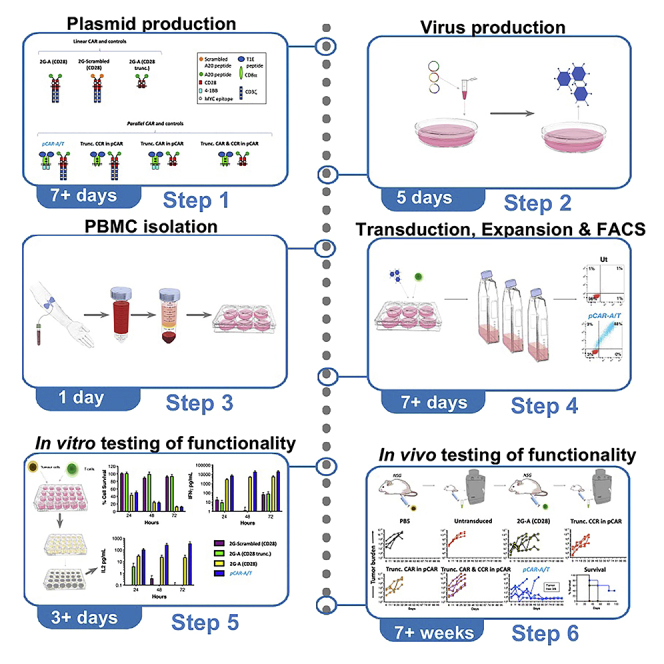

## Before you begin

### Institutional permissions

All *in vivo* experimentation must comply with local regulatory requirements. In this case, experimental work was compliant with the U.K. Home Office guidelines, as specified in project license number 70/7794 or P23115EBF. In addition, all work was approved by the King’s College London animal welfare and ethical review body (AWERB).

***Note:*** Targeting of tumor-associated αvβ6 integrin is achieved by CAR T cells that incorporate the A20FMDV2 (A20) peptide, fused to a CD28+CD3ζ endodomain (Whilding et al., 2017) (Whilding et al., 2019). This second generation (2G) CAR is referred to as 2G-A (CD28). 2G-A (CD28) is included in this protocol as a control and also serves as the CAR component within illustrative pCARs described here.

***Note:*** The T1E peptide is a pan-ErbB ligand derived from transforming growth factor-α and epidermal growth factor (Stortelers et al., 2002). When T1E is fused to CD28+CD3ζ, the resultant CAR is designated 2G-T (CD28)) (Davies et al., 2012). This CAR is currently undergoing phase 1 evaluation in patients with relapsed refractory head and neck squamous cell carcinoma (van Schalkwyk et al., 2013). The T1E peptide is included in this protocol as a targeting moiety of the chimeric co-stimulatory receptor (CCR) within the illustrative pCARs described here.

***Note:*** We have demonstrated that the pCAR platform optimally harnesses synergistic CD28 and 4-1BB co-stimulation to sustain T cell proliferation, cytokine release, cytokine signaling and metabolic fitness upon repeated stimulation with tumor antigen (Muliaditan et al., 2021).

***Note:*** This protocol describes the steps taken to engineer and validate pCARs that co-target αvβ6 and ErbB dimers. In each case, the CAR consists of 2G-A (CD28) while the CCR comprises the T1E peptide fused to a human CD8α spacer and transmembrane domain, followed by a 4-1BB or an alternative tumor necrosis factor receptor (TNFr) endodomain. Specificity of pCAR T cell activation is dependent upon binding to the CAR target antigen (in this case, αvβ6 integrin, which is tumor selective), while signaling is boosted upon engagement of ErbB dimers, which are less tumor selective.

***Note:*** Except for centrifugation and the blood draw, all steps in this protocol involving cell culture are performed in a laminar flow hood using aseptic technique.

## Key resources table

**Table:** 

REAGENT or RESOURCE	SOURCE	IDENTIFIER
**Antibodies**		

Anti-human/primate EGF biotinylated (reconstituted 1 mg/mL PBS, used 1:100 dilution)	R&D Systems	Catalog number (Cat#) BAF236; Research Resource Identifier Number (RRID#): AB_356307
PE Goat anti-mouse IgG (1:1000 dilution)	BioLegend	Cat# 405307
Streptavidin APC (1:100 dilution)	BioLegend	Cat# 405207

**Bacterial and virus strains**		

DH5α *E. coli*	New England Biolabs	Cat# c2987h

**Biological samples**		

Human blood-derived T cells	Healthy volunteers	West of Scotland Research Ethics Committee 3 reference # 18/WS/0047

**Chemicals, peptides, and recombinant proteins**		

1 kB DNA ladder	Invitrogen	Cat# 10787018
6× DNA loading dye	Thermo Fisher Scientific	Cat# R0611
LB Agar	Novagen	Cat# 71752-5
Agarose	MP Biomedicals	Cat# 02100267-CF
Ampicillin	Sigma-Aldrich	Cat# A9393
D-luciferin	Cambridge-Bioscience	Cat# B3000-1G
DMEM	BioScience	Cat# BE12-604Q
Fetal Bovine Serum	Sigma-Aldrich	Cat# F7524-500ML
Ficoll®-Paque PLUS	GE Healthcare	Cat# GE17-1440-02
GeneJuice® Transfection Reagent	Sigma-Aldrich	Cat# 70967-3
Human AB Male Serum	Sigma-Aldrich	Cat# H4522-100ML
IMDM	Thermo Fisher Scientific	Cat# 21980032
Isoflurane	Sigma-Aldrich	Cat# 792632
L-Glutamine solution	Sigma-Aldrich	Cat# G7513-100ML
Luria Broth, Miller’s modification	Fisher Bio Reagents	Cat# BP9723-500
Mouse serum	Sigma-Aldrich	Cat# M5905-500ML
MTT	Apollo Scientific	Cat# BID2165
Nuclease-free water	Invitrogen	Cat# AM9938
Phytohemagglutinin (PHA)-L	Sigma-Aldrich	Cat# 11249738001
Proleukin (aldesleukin), human recombinant interleukin (IL)-2	Clinigen Group	N/A
Restriction endonucleases	New England Biolabs	Multiple, as per this protocol
RetroNectin® recombinant human fibronectin fragment	Takara	Cat# T100B
RPMI 1640	Thermo Fisher Scientific	Cat# 11875093
SYBR Safe	Apexbio	Cat# A8743
Trypan Blue	Sigma-Aldrich	Cat# T8154
Trypsin_EDTA (0.05%) phenol red	Gibco	Cat# 25300062

**Critical commercial assays**		

Human uncoated IL-2 ELISA kit	Life Technologies Ltd	Cat# 88-7025-88
Human IFN-gamma DUOset ELISA	Bio-techne	Cat# DY285B
Monarch DNA Gel extraction kit	New England Biolabs	Cat# T1020S
Monarch Plasmid Miniprep kit	New England Biolabs	Cat# T1010S
Quick DNA Ligation kit	New England Biolabs	Cat# M2200S

**Experimental models: Cell lines**		

BxPC-3 (passage number <20 recommended)	ATCC	Cat# CRL-1687
CFPac-1 (passage number <20 recommended)	ATCC	Cat# CRL-1918
HEK293T (passage number <20 recommended)	ATCC	Cat# CRL-3216; RRID: CVCL_0063
MYC 1-9E10.2 [9E10] (passage number <20 recommended)	ATCC	Cat# CRL-1729
Panc0403 (passage number <20 recommended)	ATCC	Cat# CRL-2555
SKOV-3 (passage number <20 recommended)	ATCC	Cat# HTB-77

**Experimental models: Organisms/strains**		

Mouse: NSG: NOD.Cg-Prkdc^SCID^ Il2rg^tm1Wjl^/SzJ Age: 8–10 weeks Sex: male or female	Charles River	Strain code: 614

**Oligonucleotides**		

BD_006 - GTGGATGATGGTGCCGTTGCTC	Integrated DNA Technologies	N/A
BD_007 - GCCTCCAGCGGCGGGTCTGCAG	Integrated DNA Technologies	N/A
BD_008 - AGACCCGCCGCTGGAGGCGCTGTGCATA CCAGAG	Integrated DNA Technologies	N/A
PR_031 - TACCAAGAACAACTGGACCGACC	Integrated DNA Technologies	N/A

**Recombinant DNA**		

2G-A (CD28)	CAR Mechanics Group, King’s College London	Described in (Whilding et al., 2017)
2G-A (CD28 trunc.)	CAR Mechanics Group, King’s College London	Described in (Whilding et al., 2017)
PeqPam plasmid	Gift of Dr M Pule, University College London	N/A
*pCAR-A/T*	This manuscript	N/A
*pCAR-A/T (CD27)*	This manuscript	N/A
*pCAR-A/T (CD30)*	This manuscript	N/A
*pCAR-A/T (OX40)*	This manuscript	N/A
*pCAR-A/T (DR3)*	This manuscript	N/A
*pCAR-A/T (GITR)*	This manuscript	N/A
*pCAR-A/T (HVEM)*	This manuscript	N/A
*pCAR-H/T*	CAR Mechanics Group, King’s College London	Described in (Muliaditan et al., 2021)
RDF plasmid	Gift of Prof M Collins, University College London	N/A
pUCIDT	IDT	N/A
Trunc. CAR in pCAR	This manuscript	N/A
Trunc. CCR in pCAR	This manuscript	N/A
Trunc. CAR & CCR in pCAR	This manuscript	N/A

**Software and algorithms**		

FlowJo v.10 Software	Tree Star	https://www.flowjo.com/
Gene Designer	DNA2.0	https://www.atum.bio/resources/tools/gene-designer
Prism 9	GraphPad	https://www.graphpad.com/scientificsoftware/prism
SnapGene	GSL Biotech	https://www.snapgene.com/

**Other**		

14 mL polypropylene Falcon test tube, with snap cap	Thermo Fisher Scientific	Cat# 352059

## Materials and equipment

**Table:** R5 medium

Reagent	Final concentration	Amount
RPMI 1640	×1	470 mL
Human Serum	5%	25 mL
L-glutamine	2 mM	5 mL
**Total**	**n/a**	**500 mL**

***Note:*** Store R5 medium at 4°C for up to 2 weeks.

**Table:** I10 medium

Reagent	Final concentration	Amount
IMDM	×1	445 mL
Fetal Bovine Serum	10%	50 mL
L-glutamine	2 mM	5 mL
**Total**	**n/a**	**500 mL**

***Note:*** Store I10 medium at 4°C for up to 2 weeks.

**Table:** D10 medium

Reagent	Final concentration	Amount
DMEM	×1	445 mL
Fetal Bovine Serum	10%	50 mL
L-glutamine	2 mM	5 mL
**Total**	**n/a**	**500 mL**

***Note:*** Store D10 medium at 4°C for up to 2 weeks.

## Step-by-step method details

### Parallel CAR vector design

**Timing: 3–7 days depending upon the method used to generate the required DNA fragment for cloning**

This protocol outlines the cloning strategy used to insert one or more transgenes into the SFG γ-retroviral vector.

***Note:*** All constructs described in this manuscript are expressed using the SFG γ-retroviral vector (Rivière et al., 1995). The transgene(s) start codon coincides with the start codon of the endogenous *env* gene and expression is driven by the viral long-terminal repeat (LTR). In constructs containing more than one transgene (e.g., *pCAR-A/T*, Trunc. CCR in pCAR, Trunc. CAR in pCAR and Trunc CAR & CCR in pCAR), a sequence encoding a furin cleavage site (RRKR), short serine glycine linker ([SG]_2_) and an intervening ribosomal 2A skip peptide from the *Thosea Asigna* plant virus (T2A) (Szymczak et al., 2004) was placed between the two coding sequences.

**Figure 1:**
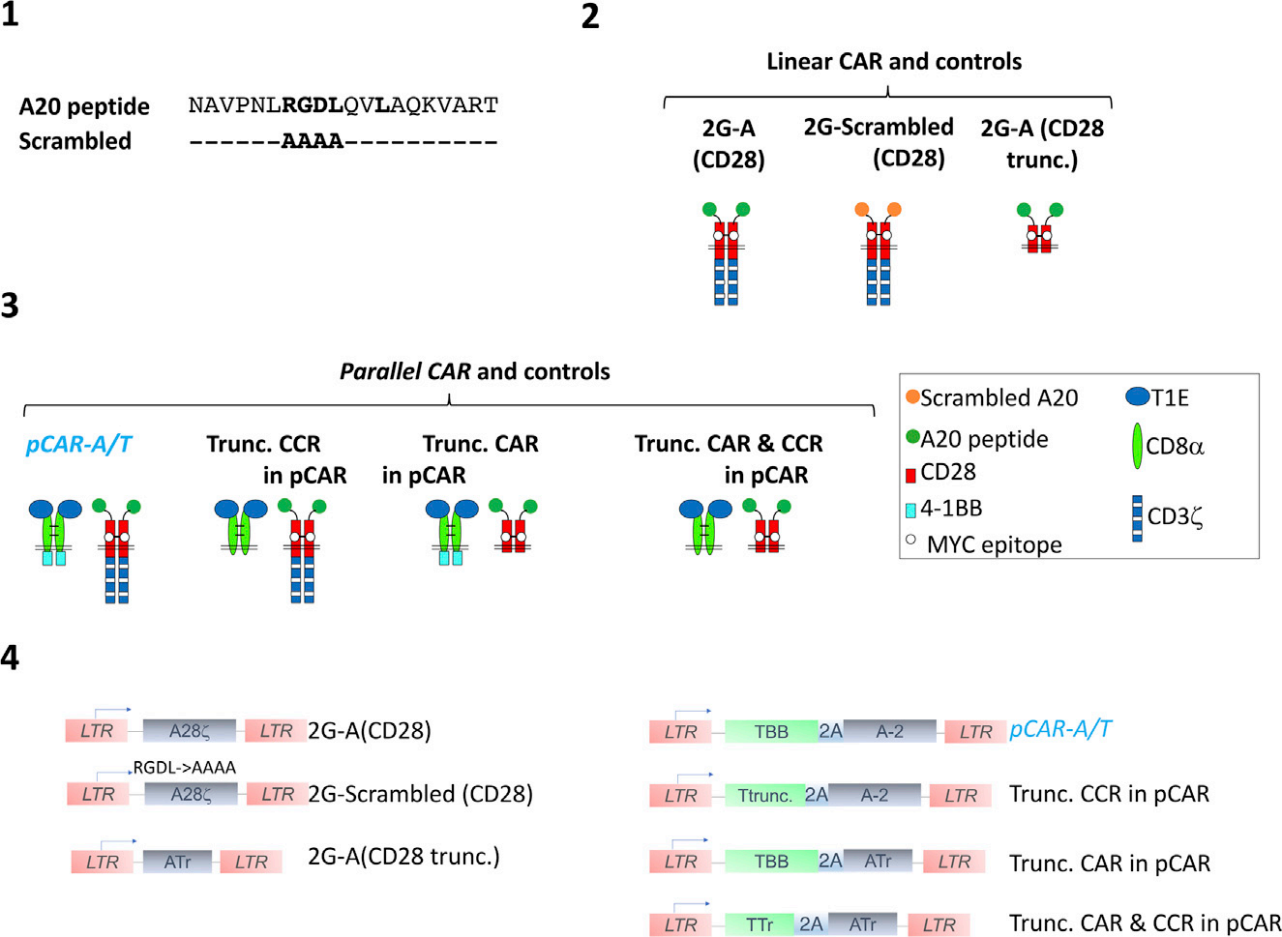
Workflow of CAR and pCAR design (1) Sequence of the A20 peptide which binds with high affinity to αvβ6 integrin. In the scrambled peptide control, the key RDGL motif within A20 has been mutated to AAAA, thereby abrogating affinity for αvβ6 integrin. (2) The A20 peptide has been introduced into a conventional CD28-containing 2G CAR to confer specificity for αvβ6 integrin (2G-A (CD28)). The scrambled peptide has been used to construct a control CAR that lacks specificity for αvβ6 integrin (2G-Scrambled (CD28)). An endodomain truncated control has also been constructed to abrogate CAR signaling capacity (2G-A (CD28 trunc.)). (3) The 2G-A(CD28) CAR has been converted to a pCAR designated *pCAR-A/T* by co-expression of a CCR in which the pan-ErbB-specific T1E peptide is coupled via a CD8α spacer and transmembrane domain to a 4-1BB endodomain. Controls have also been engineered in which the CAR or CCR endodomains have been truncated to abrogate signaling capacity (Trunc. CCR in pCAR, Trunc. CAR in pCAR and Trunc. CAR & CCR in pCAR). Note that all CARs contain an embedded MYC epitope tag within the CD28 spacer region to facilitate their detection by flow cytometry. (4) Schematic of SFG retroviral vector constructs used in this protocol. LTR – long terminal repeat.

***Alternatives:*** As an alternative to the steps described below, double stranded synthetic DNA fragments encoding the transgene to be cloned can be ordered from commercial suppliers, such as Integrated DNA Technologies (IDT – gBlock^TM^) or Genscript Inc, or they may be generated by overlap extension PCR or HiFi assembly (Xiong et al., 2006). It is advisable to codon optimize sequences using Gene Designer software for expression in the appropriate species. Alternatively, if the transgene is flanked by the appropriate restriction sites, it can be cloned directly from a donor plasmid. This approach is detailed for the generation of the *pCAR-A/T* and Trunc. CAR in pCAR constructs (Figure 1) in the step-by-step protocol provided below.

1. Linearise the SFG plasmids containing the 2G-A (CD28) and 2G-A (CD28 trunc.) constructs (Whilding et al., 2017) with *Nco*I as detailed in Table 1.

**Table 1:** Restriction digestion protocol

Reagent	Amount
Plasmid DNA	2 μg
10× NEB r3.1 buffer	5 μL
*Nco*I	2 μL (40 units)
Nuclease-free water	To a final volume of 50 μL
2. Simultaneously digest 2 μg of the *pCAR-H/T* (Muliaditan et al., 2021) with *Nco*I as detailed in Table 1.
  a. This will release a fragment referred to as TBB_T2A encoding the following elements as a single fusion polypeptide: colony-stimulating factor 1 (CSF1R) signal peptide, T1E peptide (Stortelers et al., 2003), [A]_3_ linker, amino acids 137–208 of human CD8α, amino acids 214–255 of human CD137, a furin cleavage site (RRKR), an [SG]_1_ linker (single serine followed by single glycine) and a T2A ribosomal skip peptide.
3. Incubate for 60 min at 37°C.
  a. For the 2G-A (CD28) and 2G-A (CD28 trunc.) plasmids only, dephosphorylate the 5′ ends of the linearized plasmid to avoid self-annealing of the SFG backbone. Add 2.5 μL Quick CIP, mix well and incubate for an additional 10 min at 37°C. Do not de-phosphorylate the digested *pCAR-H/T* reaction as this will prevent subsequent ligation to the linearized 2G-A (CD28)/2G-A (CD28 trunc.).
  b. Heat inactivate the *Nco*I and Quick CIP simultaneously by incubating at 80°C for 20 min.
4. Add 10 μL of 6× DNA loading dye to each 50 μL reaction and mix well.
5. Load each reaction onto a 1% agarose gel containing SYBR Safe, which is used to stain digested DNA bands. Also load a 1 kB DNA ladder to allow assessment of fragment sizes in the gel.
6. Run the gel at 100 V until a suitable separation between the required bands has been achieved.
7. Visualize the bands on an UV transilluminator and carefully cut around the required bands using a scalpel.

***Note:*** For the *pCAR-H/T,* the required TBB_T2A band should be 654 bp in size. The linearized 2G-A (CD28) and 2G-A (CD28 trunc.) bands may appear slightly bigger than the expected size of 7174 bp and 6724 bp, respectively, due to the lack of supercoiling.

**CRITICAL:** Minimize UV exposure to avoid DNA damage during fragment excision.

8. Recover the DNA fragments from the agarose gel using the Monarch DNA Gel extraction kit, following the manufacturer’s instructions, ideally eluting the DNA in 10 μL elution buffer: https://international.neb.com/products/t1020-monarch-dna-gel-extraction-kit#Protocols,%20Manuals%20&%20Usage. Accessed 27^Th^ December 2021.
9. Ligate the linearized 2G-A (CD28) or 2G-A (CD28 trunc.) plasmids with the 654 bp TBB_T2A fragment at a 1:3 molar ratio using quick ligase, as per the manufacturer’s instructions: https://international.neb.com/products/m2200-quick-ligation-kit?__cf_chl_jschl_tk__=kmegtQoKb41MzjiUyn1Cmlkgo4pFnWRnPmaChhV5CKI-1640722565-0-gaNycGzNCOU#Protocols,%20Manuals%20&%20Usage. Accessed 27^Th^ December 2021.

***Note:*** Set up a ligation reaction that lacks the presence of the TBB_T2A band to control for self-ligation of the 2G-A (CD28)/2G-A (CD28 trunc.) backbone and thus determine the efficiency of the de-phosphorylation step outlined at step 3a.

10. Following a 5 min incubation at room temperature (approximately 20°C–22°C), transform the ligation products into DH5α *E.coli* cells following the manufacturer’s instructions: https://international.neb.com/products/c2987-neb-5-alpha-competent-e-coli-high-efficiency#Protocols,%20Manuals%20&%20Usage. Accessed 27^th^ December 2021.
11. Plate onto Petri dishes coated with LB agar containing 100 μg/mL ampicillin and incubate at 37°C for 16 h.
12. Pick individual colonies in a 14 mL Falcon polypropylene tube with snap cap (see key resources table) and grow in 3 mL Luria Broth containing 100 μg/mL ampicillin for 16 h at 37°C, with agitation at 180 rpm. Snap cap tube lids should be kept loose to allow sufficient oxygenation of the culture.

***Note:*** Typically, we collect 6 colonies since this number provides a high likelihood of identifying a clone that yields the correct insert.

13. Isolate the plasmid DNA from the *E.coli* using the Monarch Plasmid Miniprep Kit, following the manufacturer’s instructions: https://international.neb.com/products/t1010-monarch-plasmid-miniprep-kit#Protocols,%20Manuals%20&%20Usage. Accessed 27^Th^ December 2021.
14. Undertake a diagnostic restriction digest aimed at confirming the successful insertion of the TBB_T2A fragment. As the TBB_T2A fragment is flanked by overhanging *Nco*I ends and can thus anneal in either orientation, select restriction enzymes that allow the orientation of the insert to be ascertained.

***Note:*** Steps 12–14 can be replaced by undertaking a colony PCR on selected colonies. Design primers to amplify over a ligation junction.

***Note:*** To determine the importance of signaling through the CCR for the improved function of pCAR T cells compared to 2G CAR T cells, two additional controls were generated, namely Trunc. CCR in pCAR and Trunc. CAR & Trunc. CCR in pCAR. To achieve this a codon-optimized (Gene Designer) double stranded DNA fragment encoding the CSF1R signal peptide, the T1E peptide, amino acids 137–206 of CD8α, a furin cleavage site, [SG]_2_ linker, T2A ribosomal skip peptide, the IL-4 receptor signal peptide, the A20FMDV peptide (shown in Figure 1) (Whilding et al., 2017) and an [A]_3_ linker was synthesized (IDT) with a 5′ *Nco*I restriction site and a 3′ *Not*I restriction site. This fragment was cloned using *Nco*I-*Not*I into 2G-A (CD28) and 2G-A (CD28 trunc.), to give Trunc. CCR in pCAR and Trunc. CAR & Trunc. CCR in pCAR respectively (Figure 1).

***Note:*** Predicted structures of CARs, pCARs and controls used in this protocol together with a schematic of accompanying SFG retroviral vectors are shown in Figure 1.

### Producing transient retrovirus

**Timing: 5 days**

**Figure 2:**
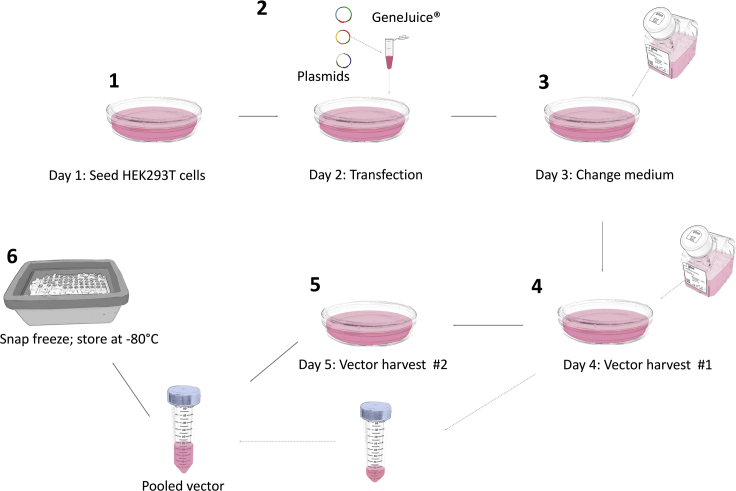
Workflow of retrovirus production (1) Seed HEK293T cells. (2) Transfection. (3) (Optional) Replace medium if a toxic transfection reagent was used. (4) Aspirate 48 h viral supernatant and store at 4°C for 18 h. Replenish medium. (5) Aspirate 72 h viral supernatant and pool with 48 h viral harvest. (6) Prepare 1.5 mL aliquots of viral supernatant and snap freeze. Store at −80°C.

The step of transfection allows the introduction of plasmid DNA into eukaryotic cells. This protocol uses GeneJuice® Transfection Reagent, a non-liposomal formulation, to introduce DNA into HEK293T cells. This method results in transient expression of the vector genome and the critical proteins (*gag, pro, pol and env)* required for the production of functional, yet replication incompetent, retroviral particles (Figure 2).

***Alternatives:*** There are other protocols available to efficiently produce retroviral particles that contain the transgene(s) of interest. Should such methods already be established in the users’ laboratory, these can be used as a suitable alternative method.

15. At least four days before the transfection:
  a. Thaw a vial of HEK293T cells for 2 min in the water bath at 37°C.
  b. Transfer the contents of the tube to 9 mL of D10 medium (pre-warmed in a 37°C water bath) and centrifuge at 400 × *g* for 5 min.
  c. Aspirate the supernatant, resuspend the cell pellet in 10 mL of D10 medium (pre-warmed in a 37°C water bath), and transfer to a 75 cm^3^ culture flask.
  d. Incubate at 37°C & 5% CO_2_.
  e. If there are significant numbers of dead cells present, gently replace the medium after 24 h with 10 mL of fresh D10 medium (pre-warmed in a 37°C water bath).
  f. Cells should be ready for passage in 2–3 days. Passage cells when at 80% confluence using the following steps.
  g. Remove and discard culture medium from HEK293T cells.
  h. Rinse the 75 cm^3^ flask with 5 mL PBS to remove residual serum and discard.
  i. Add 3 mL of Trypsin-EDTA solution and incubate at 37°C & 5% CO_2_ for 5–15 min.
  j. Confirm the cell layer has detached by looking at the cells under an inverted microscope.
  k. Add 7 mL D10 medium to inactivate the trypsin.
  l. Cells are split to a 1:10 ratio. Add 1 mL of the trypsinized cells to 9 mL fresh D10 in a new 75 cm^3^ culture flask.

***Note:*** Transfection can also be performed with HEK293T cells already in culture. However low passage number HEK293T cells more reliably produce higher viral titers following transfection. See troubleshooting 1.

***Note:*** HEK293T cells detach very easily meaning that procedures such as medium exchange must be carried out very gently.

16. One day prior to transfection, seed HEK293T cells into 10 cm^2^ tissue culture treated dishes.
  a. Remove and discard culture medium from HEK293T cells.
  b. Rinse the 75 cm^3^ flask with 5 mL PBS to remove residual serum and discard.
  c. Add 3 mL of Trypsin-EDTA solution and incubate at 37°C & 5% CO_2_ for 5–15 min.
  d. Confirm the cell layer has detached by looking at the cells under an inverted microscope.
  e. Add 5 mL of I10 medium (IMDM + 2 mM L-glutamine + 10% fetal bovine serum) and count the cells using trypan blue and a hemocytometer.
  f. Re-suspend cells to a final concentration of 1.5 × 10^5^/mL in I10 medium.
  g. Add 11 mL of cell suspension to each 10 cm^2^ culture dish (1.65 × 10^6^ cells per dish).
  h. Gently rock the dishes from side to side to aid even distribution of cells.
  i. Place dishes in an incubator at 37°C & 5% CO_2_ for 24 h.

***Note:*** Each 10 cm^2^ culture dish will produce 22 mL viral supernatant, which is enough to transduce 4 million T cells.

17. On the day of transfection, prepare as described below for each 10 cm^3^ dish of HEK293T cells.
  a. Mix 470 μL serum-free IMDM medium with 30 μL GeneJuice® in a 2 mL Eppendorf tube. GeneJuice® is a nonlipid-based transfection reagent which exhibits low levels of cytotoxicity on a wide variety of cell types.
  b. Pipette up and down and incubate at room temperature (approximately 20°C–22°C) for 5 min.
  c. Add 4.6875 μg PeqPam3 plasmid, 3.125 μg RDF plasmid and 4.6875 μg of the appropriate retroviral vector plasmid.
  d. Mix gently using pipette and incubate for 15 min at room temperature (approximately 20°C–22°C) (do not vortex).
  e. Add total volume dropwise to the HEK293T plate and swirl contents to thoroughly mix.
  f. Incubate the cells at 37°C & 5% CO_2_ for 48 h.

***Optional:*** If using alternative transfection reagents that are toxic to HEK293T cells (e.g., FuGene or PEI), change medium on HEK293T cells 24 h after transfection by adding 11 mL of fresh I10 medium pre-warmed to 37°C.

18. 48 h after transfection:
  a. Label a 50 mL Falcon tube for each transfected dish of HEK293T cells.
  b. Place Falcon tubes on ice for 5 min.
  c. Remove supernatant from HEK293T cells and place into the pre-chilled 50 mL Falcon tubes.
  d. Replenish 11 mL of I10 medium (pre-warmed to 37°C) to each dish and return to the incubator.
  e. Store the Falcon tubes containing 48 h supernatant in a 4°C refrigerator for 24 h (to be pooled with 72 h tube).

***Note:*** Since replication-defective retrovirus is present in these supernatants, they should be handled at the appropriate biosafety level (containment level 2 in our Institution).

19. 72 h after transfection:
  a. To enable snap freezing of retroviral vector-containing supernatant, prepare an ethanol bath by adding 100% ethanol into a rubber container containing a tube rack until the rack is submerged. Add dry ice to the bath until level with the ethanol.
  b. Remove 72-h supernatant from HEK293T cells and combine with 48-h supernatant on ice, filter using a 0.45 μm sterile filter (optional – please see below).
  c. Prepare 1.5 mL aliquots of supernatant into labeled tubes and put into the ethanol bath rack until completely frozen.
  d. Transfer tubes to −80°C storage as soon as possible once frozen.

***Note:*** Viral supernatant can be used fresh from harvest to transduce T cells.

**CRITICAL:** The 0.45 μm sterile filter will remove any HEK293T cells which may have detached during the supernatant harvest step. This step is critical if fresh viral supernatant is used for transduction. However, it may be omitted if virus is snap frozen since HEK293T cells will not survive this step.

***Note:*** If snap-freezing virus, it is imperative to label the tubes using printed labels. The ethanol in the freezing mix will dissolve any labeling written in permanent marker.

### Peripheral blood mononuclear cell (PBMC) isolation and activation

**Timing: 3 h**

**Figure 3:**
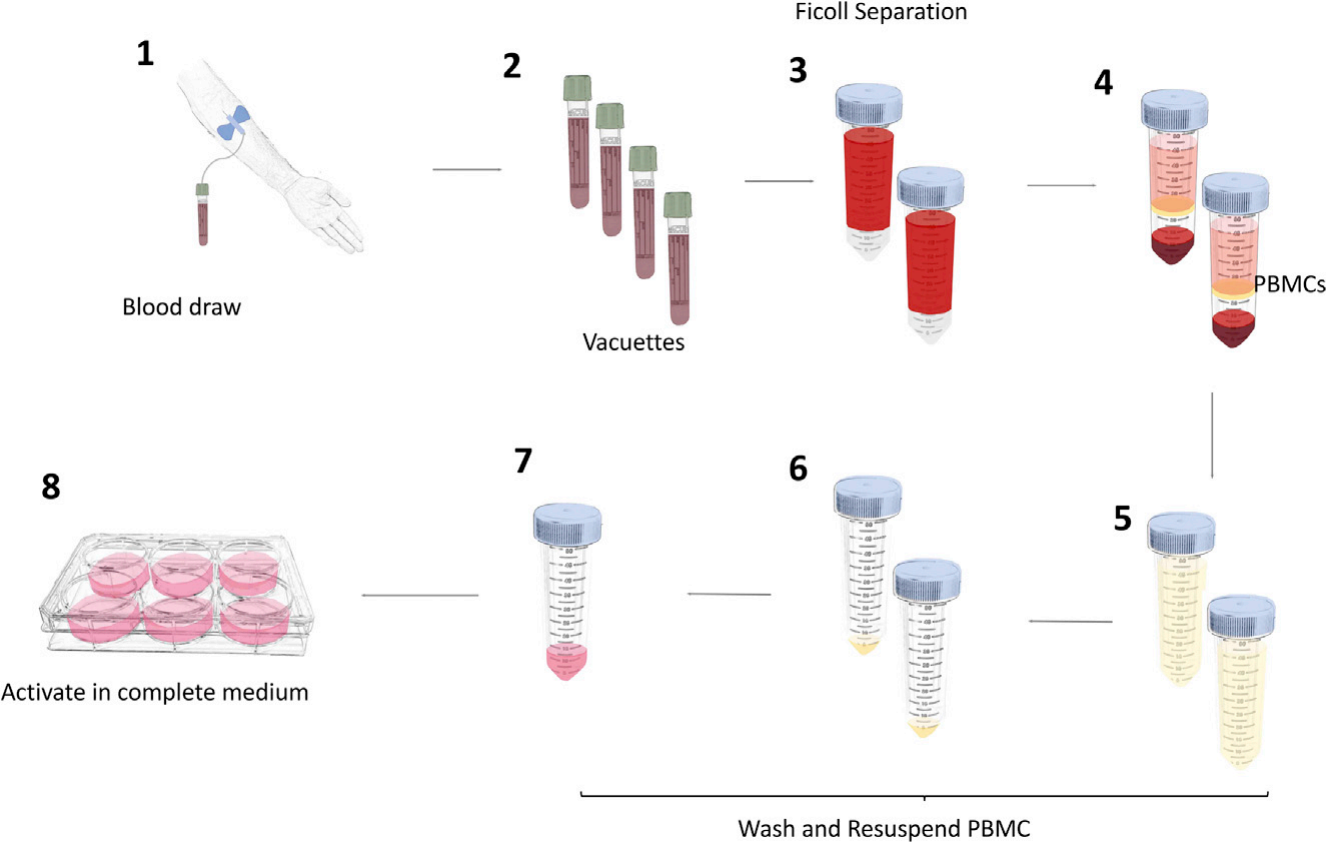
Workflow for isolation and activation of peripheral blood mononuclear cells (PBMCs) (1) Peripheral blood withdrawal. (2–4) Transfer blood onto Ficoll-Paque containing Falcon tubes for density gradient centrifugation. (5) Isolation of PBMC layer. (6–7) Washing of pooled PBMCs. (8) Activation of PBMCs using phytohemagglutinin (PHA)-L.

This method results in the generation of activated T cells that are suitable for retroviral transduction (Figure 3).

20. Peripheral blood is acquired by a trained phlebotomist or clinician from healthy consenting donors into 9 mL lithium heparin-coated vacuette tubes.
  a. Use the following equation to determine the volume of blood needed:

$$\text{Volume}\;\text{of}\;\text{ blood}\;\left(\text{mL}\right)=(\frac{\text{Number}\;\text{of}\;\text{activated}\;\text{PBMCs}\;\text{required}\;\text{at}\;\text{transduction}\;\text{step}}{1\times 10^{6}\;\text{PBMCs/mL}\;\text{blood}})\times 2$$

***Note:*** This assumes an average density of 1 × 10^6^ PBMCs per mL of blood and accounts for a contingency of a 50% reduction in PBMCs number between the activation and transduction steps.

***Note:*** All steps following the blood draw are performed in a laminar flow hood using aseptic technique.

21. Pool blood from 9 mL vacuette tubes into 50 mL Falcon tubes.
22. Wash each vacuette tube with 5 mL sterile PBS and add to the pooled blood.
23. Aliquot 15 mL Ficoll-Paque PLUS into 50 mL Falcon tubes (1 Falcon tube per 35 mL whole blood).
24. Hold the Ficoll-Paque Plus tube as close to horizontal as possible and layer 30 mL of PBS-diluted whole blood on top using a pipette aid and a 25 mL stripette.

***Note:*** Whilst layering the blood, slowly reduce the angle of the Falcon tube until it is upright.

25. Repeat step 24 until all whole blood is used.

***Note:*** Use pipette aid on lowest speed setting to layer whole blood onto the Ficoll-Paque PLUS to prevent the blood and Ficoll from mixing.

26. Centrifuge all tubes at 800 × *g* for 35 min with brake and acceleration both set to zero.
27. Using a Pasteur pipette, carefully remove the PBMC layer from the Falcon tubes and transfer into a fresh 50 mL Falcon tube.

***Note:*** Using a Pasteur pipette rather than a Gilson pipette decreases the risk of mixing layers.

**CRITICAL:** take care not to draw up Ficoll-Paque PLUS when removing the PBMC layer. Excessive Ficoll will prevent cells from pelleting during subsequent centrifugation.

28. Add sterile PBS to a final volume of 50 mL per Falcon tube.
29. Centrifuge at 400 × *g* for 10 min at room temperature (approximately 20°C–22°C).
30. Remove supernatant, being careful not to disturb the cell pellet.
31. Add 1 mL PBS to each pellet, re-suspend, and combine in a single fresh 50 mL Falcon tube.
32. Wash all tubes with 5 mL sterile PBS and combine with the previously harvested PBMCs to maximize retrieval of cells.
33. Centrifuge at 400 × *g* for 10 min at room temperature (approximately 20°C–22°C).
34. Aspirate PBS, being careful not to disturb the cell pellet.
35. Re-suspend cell pellet in 10 mL R5 medium (RPMI + 2 mM L-glutamine + 5% human male AB serum).
36. Mix 10 μL PBMC suspension with 10 μL trypan blue and count using a hemocytometer.
37. Dilute PBMCs using R5 medium to achieve a final concentration of 3 × 10^6^ cells/mL.
38. Seed cells into tissue culture-treated 6 well plates containing 4 mL per well.
39. Add phytohemagglutinin (PHA)-L to T cells to a final concentration of 5 μg/mL and mix well.

***Note:*** Numbers of PBMC between 3 – 100 million can be routinely activated in this manner. However, for scalable manufacture with a view to clinical application, alternative culture vessels and activation stimuli should be used (van Schalkwyk et al., 2021).

40. Incubate at 37°C & 5% CO_2_ for 48 h.
  a. Do not add or change medium over this period since some T cell loss typically occurs and medium is conditioned with cytokines derived from activated T cells.
  b. After 24 h, add 100 IU/mL recombinant human IL-2 to activated PBMCs.

***Optional:*** Prior to activation, T cells can be isolated from the PBMC population (for example in the event of leukemic cell contamination). Count the cells from the PBMC fraction and proceed according to the manufacturer’s protocol: (https://www.miltenyibiotec.com/US-en/products/pan-t-cell-isolation-kit-human.html#gref, accessed December 23^rd^, 2021).

***Note:*** Phytohemagglutinin works best when antigen presenting cells are present. Consequently, when dealing with purified T cells, it is recommended to use a T cell specific activation stimulus such as CD3/CD28 Human T-Activator Dynabeads^TM^ (manufacturer’s protocol: https://www.thermofisher.com/uk/en/home/references/protocols/proteins-expression-isolation-and-analysis/t-cell-activation-and-expansion/dynabeads-human-t-activator-cd3-cd28.html#prot2, accessed December 18^th^, 2021. An alternative option is T cell transact^TM^ (manufacturer’s protocol: https://www.miltenyibiotec.com/upload/assets/IM0017348.PDF, accessed December 18^th^, 2021).

### Retroviral transduction of T cells

**Timing: 2 h**

**Figure 4:**
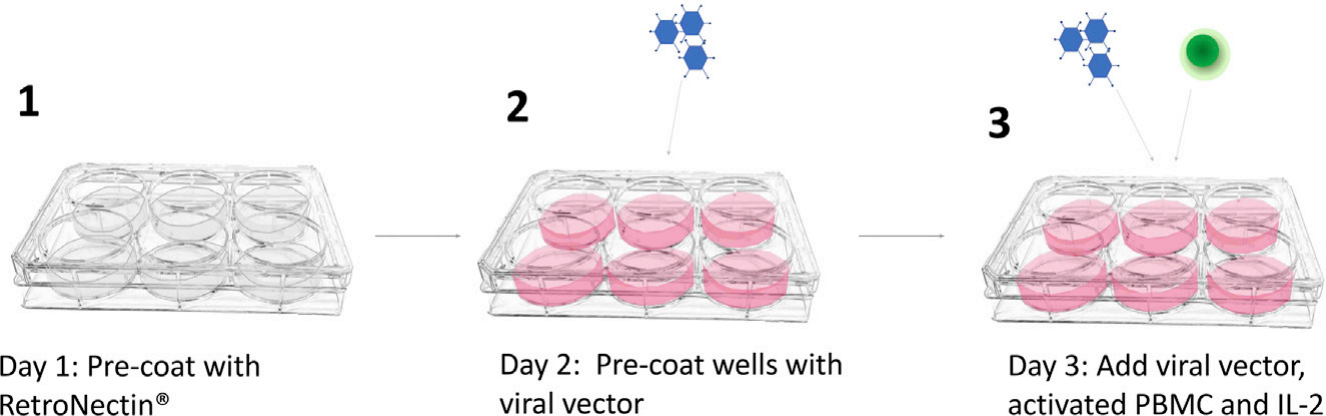
Workflow of T cell transduction (1) RetroNectin® coating. (2) Pre-load non-tissue culture (TC) treated 6 well plates with viral supernatant. (3) Add viral supernatant, IL-2 and activated T cells. Incubate for 72 h at 37°C and 5% CO_2_.

This method results in stable genetic modification of T cells to express pCARs, CARs and suitable controls (Figure 4).

41. 24 h before transduction:
  a. Add 200 μL RetroNectin® (1 mg/mL stock solution) to 18 mL sterile PBS in a 50 mL Falcon tube.
  b. Using a Pasteur pipette, aliquot 3 mL into each well of a non-tissue culture-treated 6 well plate.
  c. Wrap the 6 well plate in Saran Wrap and store for 18 h in a refrigerator at 4°C.

***Note:*** Optimally, 1 million activated PBMC are transduced in each well of the RetroNectin®-coated 6 well plate. Calculate how many wells are needed for each construct to be transduced.

**CRITICAL:** RetroNectin® can stick to certain plastics. Use polypropylene Pasteur pipettes to minimize protein loss during reagent preparation.

42. On the day of transduction:
  a. Rapidly thaw 1.5 mL aliquots of viral supernatant in a 37°C water bath for the constructs of choice.
    ***Note:*** A total of 4.5 mL viral supernatant is required per 1 × 10^6^ T cells to be transduced, of which 1.5 mL will be used to pre-load the appropriate well of the RetroNectin®-coated 6 well plate. For routine experimental work using transiently produced viral vector, titer is not determined. This is because of the relatively small volumes of virus produced and the fact that titers typically lie between 4 × 10^5^–1.0 × 10^6^ viral particles per mL. This ensures a typical multiplicity of infection of between 1.8 – 4.5.
  b. Spray cryovials containing viral supernatant with 70% ethanol and transfer into a laminar flow hood.
  c. Pre-load the RetroNectin®-coated 6 well plate with viral vector by removal of RetroNectin® from each individual well and prompt addition (to avoid drying) of 1.5 mL of thawed viral supernatant.
  d. Incubate virus coated six well plate for 2 h at approximately 20°C–22°C (or 18 h at 4°C).
  e. Retrieve activated T cells from 6 well plate and transfer into a Falcon tube. After gentle mixing by inversion, count using a hemocytometer and Trypan blue.
  f. Aspirate the 1.5 mL viral vector from the preloaded RetroNectin®-coated 6 well plate.
  g. Add 3 mL of freshly thawed viral supernatant to each well.
  h. For non-transduced control T cells, add 3 mL D10 medium (DMEM + 2 mM L-glutamine + 10% fetal bovine serum).
  i. Add 1 × 10^6^ activated T cells per well of the 6 well plate. These are taken directly from the activated PBMC culture (step 39) in the appropriate volume, without any adjustment of their concentration.
  j. Add IL-2 to each well to a final concentration of 100 IU/mL.
  k. Incubate cells at 37°C & 5% CO_2_ for 72 h.
    ***Note:*** Activated T cells should not be washed prior to transduction as this will remove potentially stimulatory factors present in the medium that they have conditioned.
    ***Note:*** Do not use all activated T cells for transduction. Untransduced T cells will later be required, for example, as a control for flow cytometry analysis and/ or functional studies.

### Expansion and assessment of transduction efficiency of pCAR T cells

**Timing: 6–14 days**

**Figure 5:**
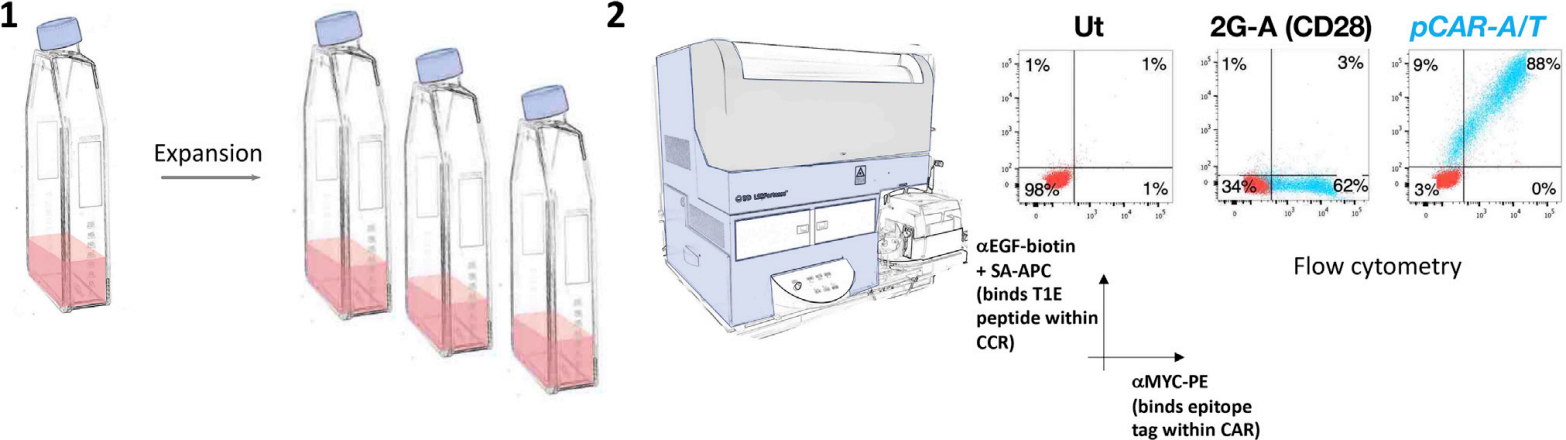
Workflow of expansion and assessment of transduction efficiency of CAR and pCAR T cells (1) T cells are supplemented with medium and IL-2 every 2–3 days to expand in culture. (2) Cell surface expression of CAR and pCAR is quantified by flow cytometry. In this representative example, CAR expression is detected by staining with 9e10 (detects a MYC epitope tag within the CAR ectodomain) while expression of the CCR component of the pCAR is detected using anti-EGF (binds to the T1E peptide in the CCR).

This method results in the production of sufficient pCAR, CAR and control T cells for functional analysis (Figure 5).

43. Every 2 days (3 days if leaving over weekends), feed T cells with 100% volume increase of R5 medium, with the addition of recombinant human IL-2 to a final concentration of 100 IU/mL.
  a. Maintain T cell concentration at below 1 × 10^6^ cells/mL.
  b. Over this interval, maintain T cells at 37°C and 5% CO_2_. See troubleshooting 2.

***Note:*** If T cell density is below 5 × 10^5^/mL, add 100 IU/mL recombinant human IL-2 only. This is because T cell growth is density-dependent, so it is undesirable to maintain cell density that is below this level.

***Note:*** Assessment of gene transfer is typically performed on day 10 post gene transfer, but it may be undertaken as early as 72 h after retroviral transduction.

44. Transfer 2.5 × 10^5^ T cells for each CAR construct into separate 5 mL flow cytometry tubes. In addition, prepare two separate tubes that contain 2.5 × 10^5^ non-transduced T cells. Wash each tube with 2 mL PBS and centrifuge at 400 × *g* for 5 min.
45. Discard PBS and resuspend the cell pellet in 50 μL PBS.
46. Leave the spare non-transduced tube without primary antibody stains.
47. Add 25 μL anti-human MYC (9e10) hybridoma supernatant to all tubes containing transduced T cells and one non-transduced tube and place on ice for 30 min.

***Note:*** 9e10 antibody is also available from commercial suppliers and may be used for this step following appropriate titration.

48. Wash each tube with 2 mL PBS and centrifuge at 400 × *g* for 5 min.
49. Discard PBS and add fresh 50 μL PBS to each tube.
50. Add 3 μL goat anti-mouse IgG PE conjugated antibody to each tube and place on ice for 30 min in the dark.

***Note:*** Anti-mouse IgG derived from other species and conjugated with an alternative fluorochrome may be used for this step.

51. Wash each tube with 2 mL PBS and centrifuge at 400 × *g* for 5 min.
52. Discard PBS and add fresh 50 μL PBS to each tube.
53. Add 10 μL mouse serum (diluted 1:10 in PBS) and incubate at room temperature (approximately 20°C–22°C) for 30 min in the dark.

***Note:*** This step serves to block any free binding sites in the goat anti-mouse IgG PE conjugated antibody added in step 50. Otherwise, there is a risk that some antibodies that are added later may bind to these unoccupied sites, leading to a spurious positive signal.

54. Add 3 μL biotinylated anti-human EGF to each tube and place on ice for 30 min in the dark.
55. Wash each tube with 2 mL PBS and centrifuge at 400 × *g* for 5 min.
56. Discard PBS and add fresh 50 μL PBS to each tube.
57. Add 3 μL SA-APC to each tube and incubate on ice for 30 min in the dark.
58. Wash each tube with 2 mL PBS and centrifuge at 400 × *g* for 5 min and return to ice.
59. Discard PBS and resuspend cells in 250 μL PBS.
60. Analyze using flow cytometry.

***Note:*** Anti-EGF is used to detect the T1E peptide on the CCR and endodomain truncated control. 9e10 antibody is used to detect the human MYC epitope tag within the 2G-A (CD28) CAR and endodomain or ectodomain controls.

***Optional:*** Gates are set using non-transduced cells that have been stained with the same reagents. However, fluorescence minus one (FMO) controls generated using transduced cells may alternatively be used to determine where to set gates.

***Optional:*** Additional T cell markers and a viability dye can be added to the staining panel to aid gating on live T cells.

### *In vitro* cytolytic activity and cytokine release by pCAR T cells

**Timing: 5 days**

**Figure 6:**
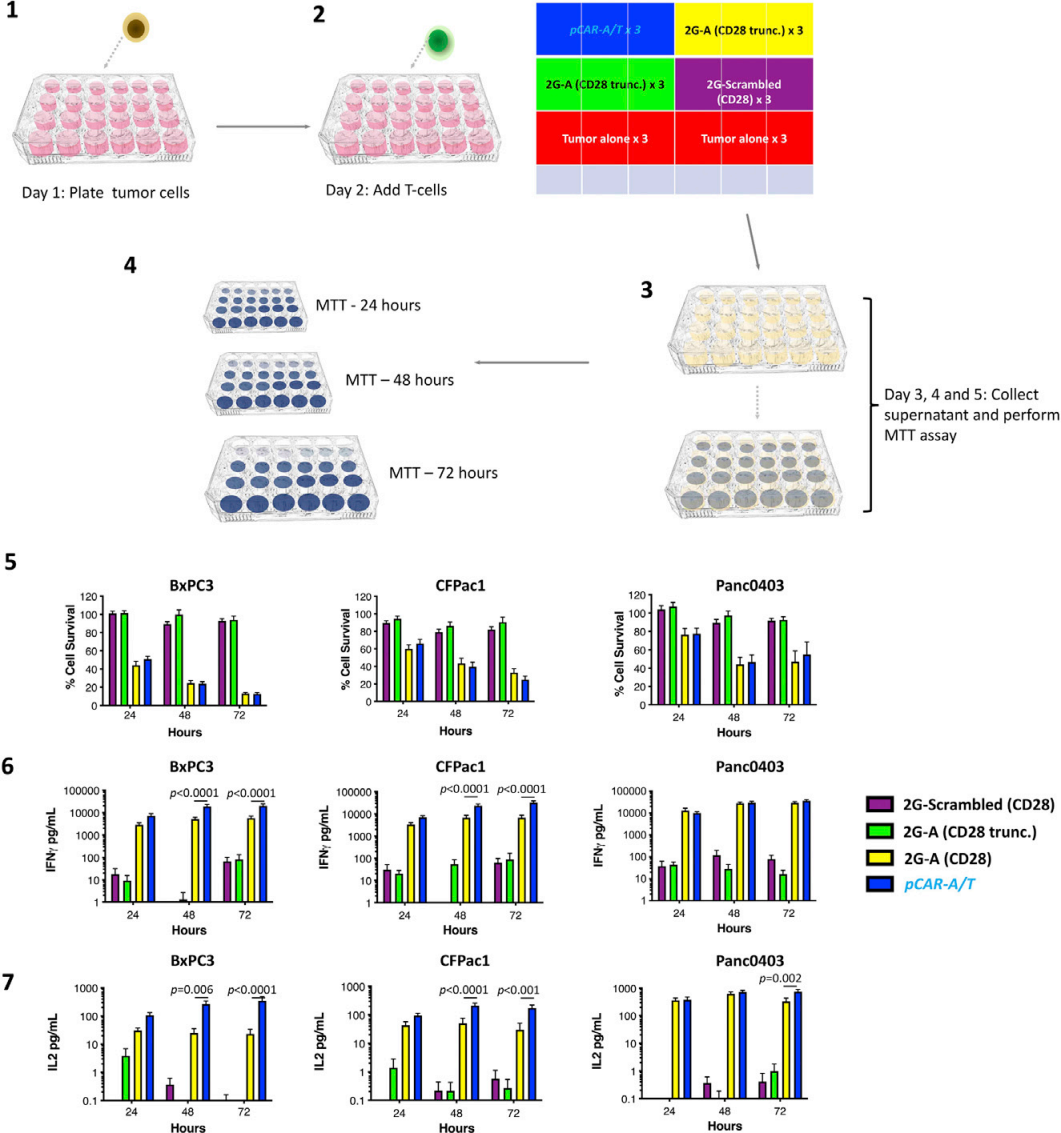
*In vitro* assessment of cytolytic activity and cytokine production by pCAR T cells (1) Seed tumor cells in three replicate 24 well plates for analysis at 24, 48 and 72 h after pCAR T cell addition. (2) After 24 h, add 1 × 10^5^ pCAR or control T cells to triplicate wells of tumor cells according to the indicated matrix. (3) Harvest supernatant at each time point for ELISA measurement of IL-2 and IFN-γ. (4) Perform MTT assay at each time point to determine residual tumor cell viability. (5) Cytolytic activity of *pCAR-A/T* and control T cells against the indicated tumor cell lines (mean ± SEM, n=5–7). (6) IFN-γ release (mean ± SEM, n=6–9) and (7) IL-2 release (mean ± SEM, n=4–5) by tumor-activated *pCAR-A/T* and control T cells. Statistical analysis was by one-way ANOVA with multiple comparisons. Comparison between *pCAR-A/T* and 2G-A(CD28) T cells is shown.

This method demonstrates the kinetics of tumor cell killing and cytokine production by pCAR T cells (24–72 h post activation), making comparison with 2G CAR T cells and controls that are predicted to lack specificity for αvβ6 integrin or lack signaling capacity (Figure 6).

***Note:*** It is necessary to validate tumor cell surface expression of the target antigen(s) recognized by the pCAR under study prior to co-culture experiments. In this example, BxPC3, CFPac-1 and Panc0403 pancreatic tumor cells have been used, all of which co-express αvβ6 integrin and multiple ErbB homo- and heterodimers.

61. Day 1 – prepare tumor cells:
  a. Seed tumor cells at 1 × 10^5^ cells in 500 μL D10 medium per well in 3 × tissue culture treated 24 well plates.
  b. Incubate cells at 37°C & 5% CO_2_ for 24 h.
62. Day 2 – prepare T cells: See troubleshooting 3.
  a. Count CAR and pCAR T cells. Re-suspend each to a final concentration of 1 × 10^5^ transduced T cells/mL in R5 medium. Prepare untransduced T cells at an equivalent density per mL to the highest density of total T cells present in the CAR and pCAR T cell tubes.
  b. Aspirate medium from the tumor cells, being careful not to disturb the monolayers.
  c. Add 1 mL CAR, pCAR or non-transduced T cells per well of tumor cells in triplicate. Add 1 mL of R5 medium to three wells to act as tumor alone control.
  d. Incubate plate 37°C & 5% CO_2_.
  e. Repeat steps 62–62 to produce three identical co-culture 24 well plates (one each for 24-h, 48-h and 72-h analysis).

***Note:*** No cytokine support is provided for T cells during the co-culture assay.

63. Day 3,4 and 5 – analyze cytotoxicity.
  a. Transfer 150 μL supernatant from each well into a labeled u-bottom 96 well plate and store at −20°C for cytokine analysis.
  b. A stock solution of MTT (5 mg/mL in PBS) is diluted 1:10 in R10 medium to give a final concentration of 500 μg/mL (13 mL/24 well plate).
  c. Carefully remove the remaining medium from each well of one 24 well plate (leaving two plates for 48 h and 72-h analysis).
  d. Pipette 500 μL of 500 μg/mL MTT solution into each well.
  e. Incubate at 37°C & 5% CO_2_ for 1 h.
  f. Aspirate MTT solution from each well being careful not to disturb the monolayers.
  g. Add 500 μL DMSO into each well.
  h. Gently swirl the plate to solubilize the formazan crystals.
  i. Read absorbance at 560 nm using a plate reader.
  j. Calculate residual tumor cell viability using the following equation:

(Absorbance of tumor + T cells/absorbance of tumor + medium control) × 100.

64. Day 3,4 and 5 – analyze cytokine production.
  a. The concentration of IL-2 and IFNγ in harvested supernatants is assessed using enzyme-linked immunosorbent assay (ELISA).
  b. Manufacturer’s protocols are followed to analyze the 150 μL aliquots of supernatant harvested in step 63a.

***Note:*** Using the methods and kits described above, supernatants for the assessment of IL-2 are diluted 1:5 in reagent diluent. Supernatants for the assessment of IFN-γ are diluted 1:10 in reagent diluent. However, it should be noted that alternative dilutions may be required using other ELISA kits in order to ensure that optical density readings lie on the linear part of the standard curve.

***Note:*** In this example, *pCAR-A/T* T cells demonstrate broadly similar *in vitro* cytolytic activity to 2G-A (CD28) CAR T cells. Cytokine release by pCAR T cells is generally greater than that seen using 2G CAR T cells (Muliaditan et al., 2021). Function of the CAR is dependent upon the intact A20 peptide and signaling endodomain, indicated by the lack of activity of the 2G-Scrambled (CD28) and 2G-A (CD28 trunc.) controls.

### *In vitro* pCAR T cell tumor re-stimulation assay

**Timing: Undetermined due to unknown persistence of Tcell restimulation capacity**

**Figure 7:**
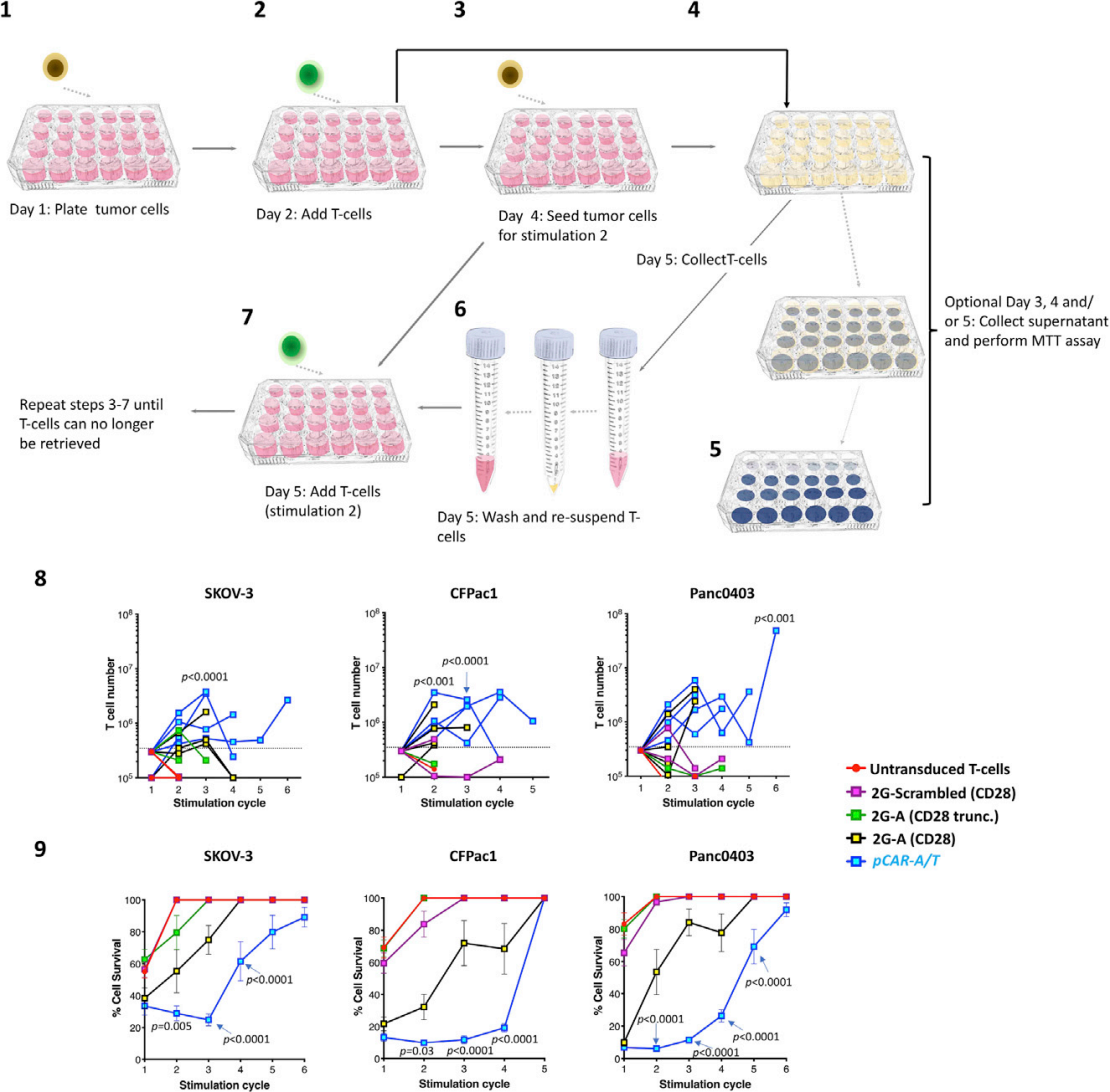
Workflow for *in vitro* pCAR T cell tumor re-stimulation assay (1) Seed tumor cells on day 1. (2) On day 2, add 1 × 10^5^ pCAR or control T cells to tumor cells. (3) On day 4, seed tumor cells for stimulation cycle 2. (4) On days 3, 4 and/ or 5, supernatant may optionally be collected for ELISA from stimulation cycle 1. (5) Perform MTT assay on day 5 to determine tumor cell viability (stimulation cycle 1). (6) Retrieve and pool pCAR and control T cells from stimulation 1 well plate. (7) Plate retrieved pCAR and control T cells on tumor cells in stimulation cycle 2 plate. Repeat steps 3–7 until T cells can no longer be retrieved from tumor monolayers. (8) Re-stimulation of *pCAR-A/T* and control T cells on the indicated tumor cell lines. T cell number (n=4; all replicates shown) and tumor cell viability (9) was at the end of each stimulation cycle (mean ± SEM, n=9–12). Statistical analysis in 8 and 9 was performed using two-way ANOVA, showing comparison between *pCAR-A/T* and 2G-A (CD28) T cells at the indicated stimulation cycle.

In cancer, persistent antigen exposure leads to progressive T cell dysfunction (Wherry and Kurachi, 2015), a process that can be modeled using tumor re-stimulation assays (Vardhana et al., 2020). This method quantifies T cell expansion, tumor cell killing and (optionally) cytokine production by pCAR T cells following iterative stimulation on tumor cells that express cognate antigen (Figure 7). In this example, twice weekly stimulation cycles are undertaken. Timing may be adjusted to allow for weekends (e.g., alternating 3 day and 4 day stimulation cycles). Alternatively, weekly stimulation cycles may be undertaken although this typically extends the duration of the experiment. Comparison is made with 2G CAR T cells and controls that are predicted to lack specificity for CAR target antigen (in this example, αvβ6 integrin) or signaling capacity.

***Note:*** First, validate tumor cell surface expression of the target antigen(s) recognized by the CAR or pCAR under study prior to co-culture experiments. In this example, SKOV-3 ovarian tumor cells and CFPac-1 and Panc0403 pancreatic tumor cells have been used. All three express high levels of αvβ6 integrin and multiple ErbB homo- and heterodimers.

65. Day 1 – prepare tumor cells:
  a. Seed tumor cells at 1 × 10^5^ cells in D10 medium per well in a tissue culture treated 24 well plate in a total volume of 500 μL per well.
  b. Incubate cells at 37°C & 5% CO_2_ for 24 h.
66. Day 2 – prepare T cells and establish co-cultures:
  a. Count CAR and pCAR T cells. Re-suspend each to a final concentration of 1 × 10^5^ transduced T cells/mL in R5 medium. Prepare untransduced T cells at an equivalent density per mL to the highest density of total T cells present in the CAR and pCAR T cell tubes.
  b. Aspirate medium from the tumor cells, being careful not to disturb the monolayers.
  c. Add 1 mL CAR, pCAR or non-transduced T cells per well of tumor cells in triplicate. Add 1 mL of R5 medium to three wells to act as tumor alone control.
  d. Incubate plate 37°C & 5% CO_2_.
67. Day 3–4 – (Optional).
  a. Harvest 150 μL supernatant from each well cytokine analysis by ELISA. Transfer into a labeled u-bottom 96 well plate and store at −20°C.
68. Day 4 – prepare tumor cells for stimulation cycle 2:
  a. Seed tumor cells at 1 × 10^5^ cells per well in a tissue culture treated 24 well plate in a total volume of 500 μL per well.
  b. Incubate cells at 37°C & 5% CO_2_ for 24 h.

69. Day 5 – Analyze stimulation cycle 1.
  ***Optional:*** Transfer 150 μL supernatant from each co-cultivation well into a labeled u-bottom 96 well plate and store at −20°C.
  a. Prepare MTT to a concentration of 500 μg/mL (13 mL/24 well plate).
  b. Carefully remove medium from each well of one 24 well plate. Pool triplicate wells into single 15 mL Falcon tubes.
  c. Wash wells with 1 mL PBS and transfer to corresponding 15 mL Falcon tubes to ensure maximum retrieval of T cells.
  d. Pipette 500 μL of 500 μg/mL MTT solution into each well.
  e. Incubate 24 well plate at 37°C & 5% CO_2_ for 1 h.
  f. Centrifuge 15 mL Falcon tubes containing T cells at 400 × *g* for 5 min.
  g. Aspirate supernatant, being careful not to disturb the cell pellet.
  h. Resuspend cells in 3.2 mL R5 medium.
  i. Aspirate medium from tumor cells seeded on day 4 (step 68).
  j. Add 1 mL of T cells to corresponding wells and incubate plate at 37°C & 5% CO_2_ (stimulation 2).
  k. With the remaining 200 μL cell suspension count using trypan blue.
  l. Aspirate MTT solution from each well of stimulation cycle 1 plate being careful not to disturb the monolayers.
  m. Add 500 μL DMSO into each well.
  n. Gently swirl the plate to solubilize the formazan crystals.
  o. Read absorbance at 560 nm using a plate reader.
  p. Calculate residual tumor cell viability using the following equation:
    (Absorbance of tumor + T cells/absorbance of tumor + medium control) × 100.
  q. Repeat steps 65–69 twice per week (e.g., preparing tumor cells on day 1, day 5, day 8, day 12 and T cells on day 2, day 6, day 9, day 13 etc.) until T cells can no longer be retrieved at the end of a stimulation cycle.
    ***Note:*** Parallel CAR T cells can generally be re-stimulated on tumor monolayers through a greater number of cycles than linear CAR T cells. They also maintain cytolytic activity through a greater number of stimulation cycles owing to their greater resistance to tumor-induced T cell exhaustion.

### *In vivo* assessment of CAR T cell functionality

**Timing: 5–16 weeks**

**Figure 8:**
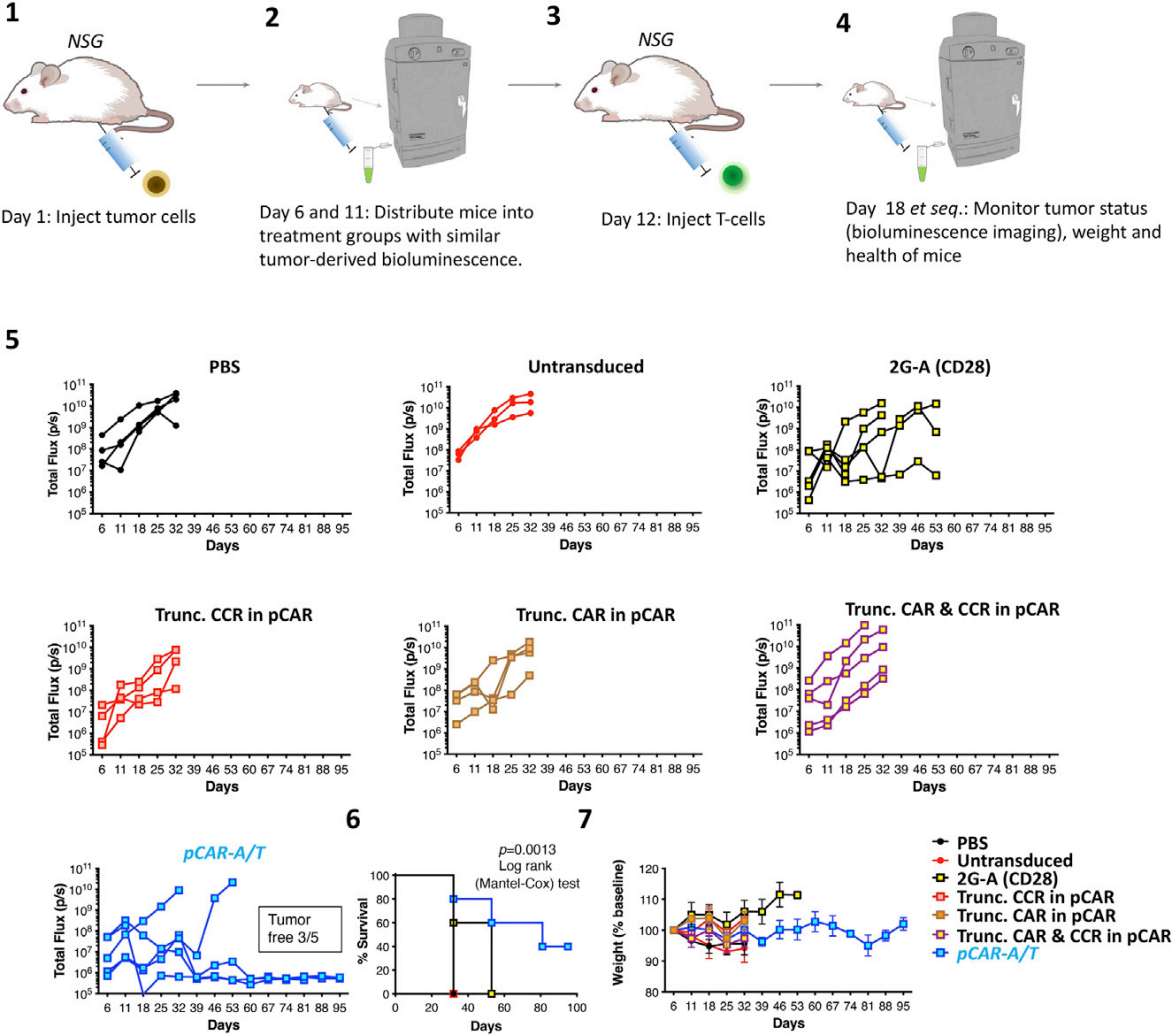
Workflow for evaluation of *in vivo* anti-tumor activity of pCAR T cells (1) Tumor injection into NSG mice. (2) Bioluminescence imaging to confirm tumor engraftment and distribution of mice into treatment groups with similar mean tumor burden. (3) Injection of pCAR, CAR and control T cells or PBS. (4) Serial BLI to monitor tumor status. (5) Anti-tumor activity of *pCAR-A/T* and control T cells in NSG mice with an established BxPC3 tumor burden. T cell administration was performed 12 days after tumor cell inoculation. Tumor burden in individual mice following treatment is monitored by BLI. (6) Kaplan Meier survival curves and (7) weight of treated mice (mean ± SEM).

This method quantifies *in vivo* efficacy of pCAR T cells in a challenging BxPC3 intraperitoneal (i.p.) xenograft model of pancreatic cancer (Figure 8). Tumor cells co-express red fluorescent protein (RFP) and firefly luciferase (ffLuc), achieved by retroviral transduction and flow sorting to purity (Whilding et al., 2017). Comparison is made with the second generation 2G-A (CD28) CAR which has limited efficacy in this model (Whilding et al., 2017). A series of pCAR controls is also included in which binding to both αvβ6 integrin and ErbB dimers is retained, but in which the CAR, CCR or both signaling endodomains have been truncated to disable function.

***Note:*** Group sizes should be calculated in advance of undertaking the experiment. A minimum group size of 5 mice per group can provide useful information and is convenient since 5 animals can be subject to BLI simultaneously. However, this design may compromise power, especially in the event of incidental loss of one or more mice during the experiment. If variability (e.g., standard deviation) of tumor growth are known, a power calculation can be performed (strongly advised), for example using http://www.biomath.info/power/ttest.htm (accessed March 12^th^, 2022). See troubleshooting 4.

***Note:*** To minimize risk of bias, it is recommended that animal work is undertaken in a blinded fashion (e.g., where one staff member prepares pCAR, CAR and control T cells as coded samples for administration by a second staff member who is unaware of which treatment has been administered to each group). Treatment groups and their order of treatment should also be randomly assigned.

70. Day -2 – Isolation and activation of PBMCs.
  a. Perform steps 21–61 to expand sufficient numbers of transduced T cells for each treatment group. Non-transduced control T cells should also be retained.

***Note:*** CAR/ pCAR T cell expansion data from previous experiments is required to calculate the number of T cells required for transduction of each construct. In general, at least 10 million transduced cells can be generated from 1 million transduced activated T cells over 10 days when validated viral vector is used for the gene transfer step.

71. 6–10-week-old male or female NSG mice are purchased from Charles River Laboratories.

***Note:*** Adequate time must be allowed for mice to acclimatize between delivery and start of experiments (generally 7 days).

72. Day 1 – Establishment of BxPC3 human pancreatic ductal adenocarcinoma xenograft in NSG mice.
  a. On the day of tumor injection, take baseline weight readings and check mice for signs of ill health.
  b. Inject 1 × 10^5^ ffLuc/RFP-expressing BxPC3 tumor cells suspended in 100 μL sterile PBS into all mice using the i.p. route.
  c. Weigh mice twice weekly and check for signs of ill health. Weight loss greater than 15% caused by tumor burden is a humane endpoint and mice must be sacrificed.

***Note:*** Mice should also be humanely killed using an appropriate procedure (referred to in the UK as a Schedule one procedure; https://www.legislation.gov.uk/ukpga/1986/14/schedule/1, accessed January 2^nd^, 2022) if the tumor total flux reading equals or exceeds 1 × 10^10^, or if the tumor ulcerates, limits normal behavior, impedes a vital function (e.g., locomotion, vision, mastication, excretion etc.), or causes clinical signs such as jaundice. Increase in body girth (similar to a pregnant mouse), dyspnoea, neurological signs, lameness or general signs of ill-health (e.g., piloerection, hunched posture, inability to groom, inactivity, inappetance) are all additional humane end points.

73. Day 1 (continued) – Transduce T cells.
  a. Perform steps 20–60 to generate sufficient numbers of pCAR, CAR and control T cells for each experimental group.
74. Day 6 – Bioluminescence imaging of mice.
  a. Mice are injected i.p. with 150 mg/kg D-luciferin.
  b. Place mice into an anesthetic induction chamber (isoflurane 2.5%, with flow rate 2Lmin) until fully anesthetized.
  c. Precisely 15 min after the D-luciferin injection, using an IVIS Spectrum Imaging platform (Perkin Elmer) with Living Image software, image the mice using the auto-exposure function.
  d. Total flux (photons/second) is determined by creating a region of interest over each entire mouse.
  e. After imaging, return the mice to their cages and monitor until they have fully regained consciousness.

**CRITICAL:** Prior to starting experiments, follow Perkin Elmer protocols to determine the D-luciferin kinetic curve for your model (https://resources.perkinelmer.com/lab-solutions/resources/docs/SOP_Determine_Luciferin_Kinetic_Curve.pdf, accessed January 2^nd^, 2022). Because bioluminescence signal production can be tissue dependent, Perkin Elmer recommend optimal timings between injection of D-luciferin substrate and imaging is determined prior to starting experiments. Serial imaging is performed every 5 min until 40 min post substrate injection. Total flux is plotted against time and this curve is used to determine an imaging time point to produce maximum total flux (usually between 10–20 min post injection).

75. Day 11 – Bioluminescence imaging of mice.
  a. Mice that have total flux values greater than the enrollment threshold (usually >1 × 10^7^ photons/second) are distributed into treatment groups with similar average tumor burden.
76. Day 12 – Administration of T cells and PBS.
  ***Note:*** Each mouse should receive the appropriate dose of transduced T cells (4 × 10^6^ cells in this example). If the transduction efficiency is lower than 100%, the total number of T cells is adjusted to bring the transduced population up to 4 × 10^6^ cells. Illustrating this, if the transduction efficiency is 66.6%, 6 × 10^6^ total T cells would be injected into each mouse.
  a. Count transduced and non-transduced T cells.
  b. Prepare transduced T cells in PBS at a concentration of 2 × 10^7^ transduced T cells/mL.
  c. Weigh mice.
  d. Inject 200 μL of either pCAR, CAR or non-transduced T cells i.p. into the mice.
  e. Inject 200 μL PBS into control mice.
  f. If untransduced T cells are used as an additional control, infuse the same total T cell number in this group as was employed in the pCAR or CAR treatment group that received the highest total T cell number.
77. Mice are monitored daily for signs of ill health and weighed twice weekly. Reduced weight, piloerection, ruffled coat, poor appetite, and reduced locomotion can all be signs of graft versus host disease (GvHD). Animals should be humanely killed if they show these signs without improvement for 48 h; if they lose >15% of body weight or if they develop other GvHD related signs such as diarrhea (>48 h).
78. To monitor tumor status, weekly bioluminescence imaging (BLI) is performed for the duration of the study.
79. Mice with total flux (p/s) values reaching the humane endpoint threshold (1 × 10^10^ p/s) are humanely sacrificed by cervical dislocation or asphyxiation in CO_2_. Tumors are fixed in 10% formalin and paraffin embedded for future analysis.

***Note:*** Parallel CAR T cells manifest enhanced *in vivo* anti-tumor activity compared to linear CAR cells. An important underlying mechanism is superior functional persistence of pCAR T cells *in vivo* (Muliaditan et al., 2021).

### *In vitro* evaluation of pCARs that utilize alternative members of the tumor necrosis receptor family

**Timing: Undetermined due to unknown persistence of Tcell restimulation capacity**

**Figure 9:**
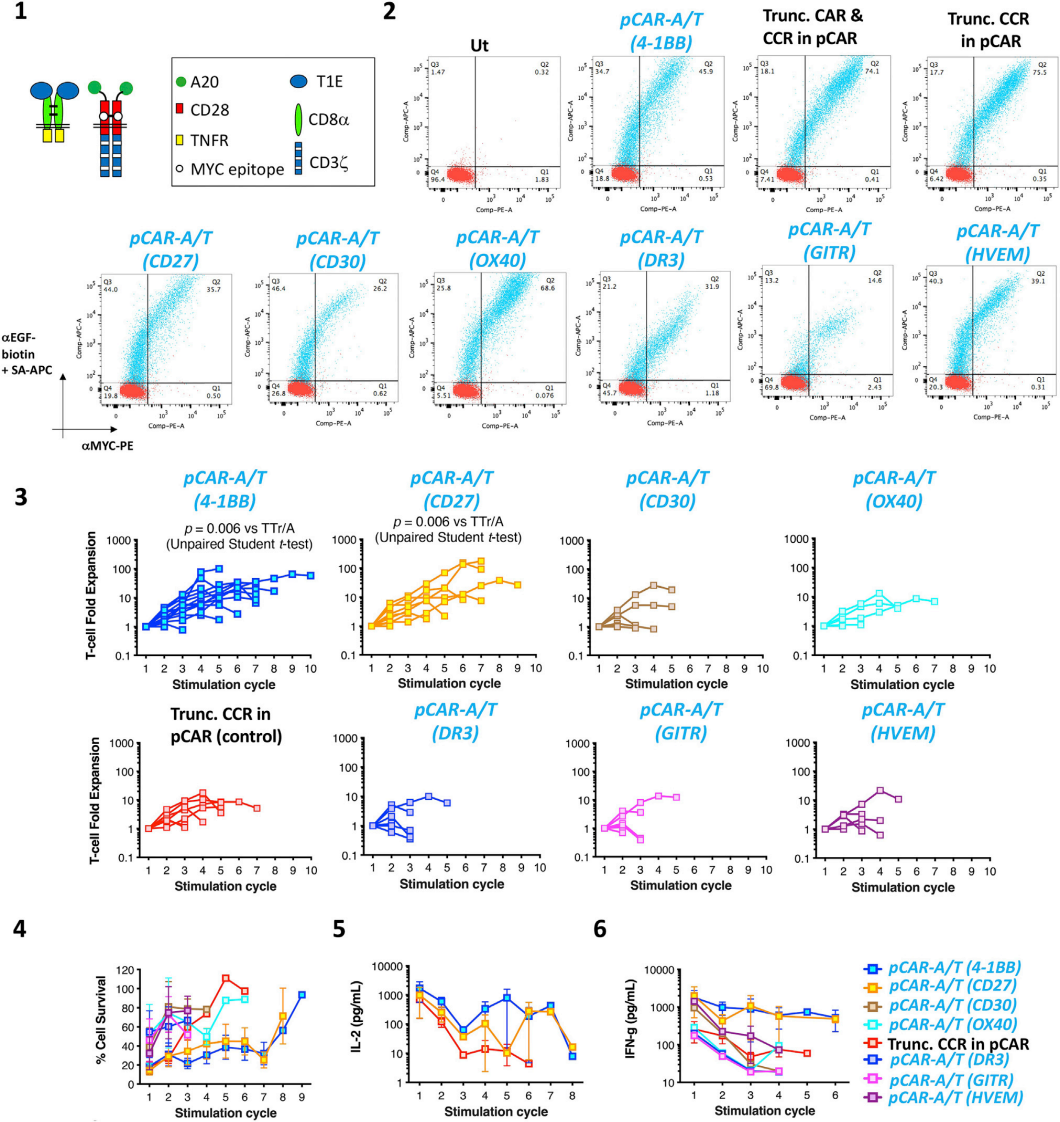
Workflow for comparison of pCARs containing alternative TNFr modules (1) Generic pCAR structure. (2) Flow cytometric analysis of pCAR expression. In these representative examples, CAR expression is detected after staining with 9e10 (detects a MYC epitope tag within the CAR ectodomain) while expression of the CCR component of the pCAR is detected using anti-EGF (binds to the T1E peptide in the CCR). (3) Fold expansion of pCAR and control T cells in weekly re-stimulation cycles on BxPC3 tumor cells. (4) Tumor cell viability was assessed 48 h after initiation of each stimulation cycle (mean ± SEM). (5) IL-2 and (6) IFN-γ was measured in supernatant collected 24 h after initiation of each stimulation cycle (Mean ± SEM).

All pCARs described above and in our earlier work (Muliaditan et al., 2021) deliver co-stimulation by CD28 and the tumor necrosis factor receptor (TNFr) family member, 4-1BB. The method described here compares *in vitro* anti-tumor activity of this pCAR with alternatives in which CD28 is paired with another TNFr family member (Figure 9). Specifically, the 4-1BB endodomain within *pCAR-A/T* CCR has been replaced by CD27, CD30, OX40, DR3, GITR or HVEM. The *pCAR-A/T* pCAR is used as positive control, given its superior anti-tumor activity compared to 2G CARs and other controls. The Trunc. CCR in pCAR construct is used as negative control since it retains full ligand binding activity but lacks a functional TNFr endodomain. Tumor re-stimulation assays were used to make this comparison in the example shown, although any of the other *in vitro* or *in vivo* assays could similarly be employed.

80. Clone alternative TNFr domains into SFG plasmid vector encoding *pCAR-A/T.*
  a. DNA fragments were synthesized as codon optimized sequences inserted in the pUCIDT plasmid (IDT).
  b. Fragments encoded amino acids 137–206 of CD8α, fused in frame with either amino acids 213–260 of CD27, amino acids 408–595 of CD30, amino acids 216–277 of CD40, amino acids 221–417 of DR3, amino acids 184–241 of Glucocortocoid-induced TNFR-related protein (GITR) or amino acids 224–283 of Herpes virus entry mediator (HVEM). The fragments also encoded for a furin cleavage site (RRKR), [SG]_2_ linker, T2A ribosomal skip peptide, the IL-4 receptor signal peptide, the A20FMDV peptide (Whilding et al., 2017), an [A]_3_ linker and amino acids 114–134 CD28 (in which amino acids 117–122 had been replaced with the myc tag sequence).
  c. These fragments were amplified by PCR using primers BD_008 and BD_006 (see key resources table).
  d. Products were fused by overlap extension PCR to a secondary fragment generated from pCAR-A/T using primers PR_031 and BD_007 (see key resources table).
  e. The resulting PCR products were cloned into pCAR-A/T as an *Nco*I-*Not*I fragment.
81. General retroviral vector for all new pCARs using methods described in steps 15–19.
82. Isolate and activate T cells as described in steps 20–40.
83. Transduce activated T cells as described in steps 41–42.
84. Expand and analyze T cells for pCAR expression as described in steps 43–60.
85. Tumor re-stimulation assays were performed on BxPC3 tumor cells as described in steps 65–69.

***Note:*** Tumor re-stimulation activity of pCAR T cells that contain CD28 and either 4-1BB or CD27 is most sustained, when compared to alternative TNFrs. This is accompanied by a trend towards more sustained cytokine release and tumoricidal activity.

## Expected outcomes

**Figure 10:**
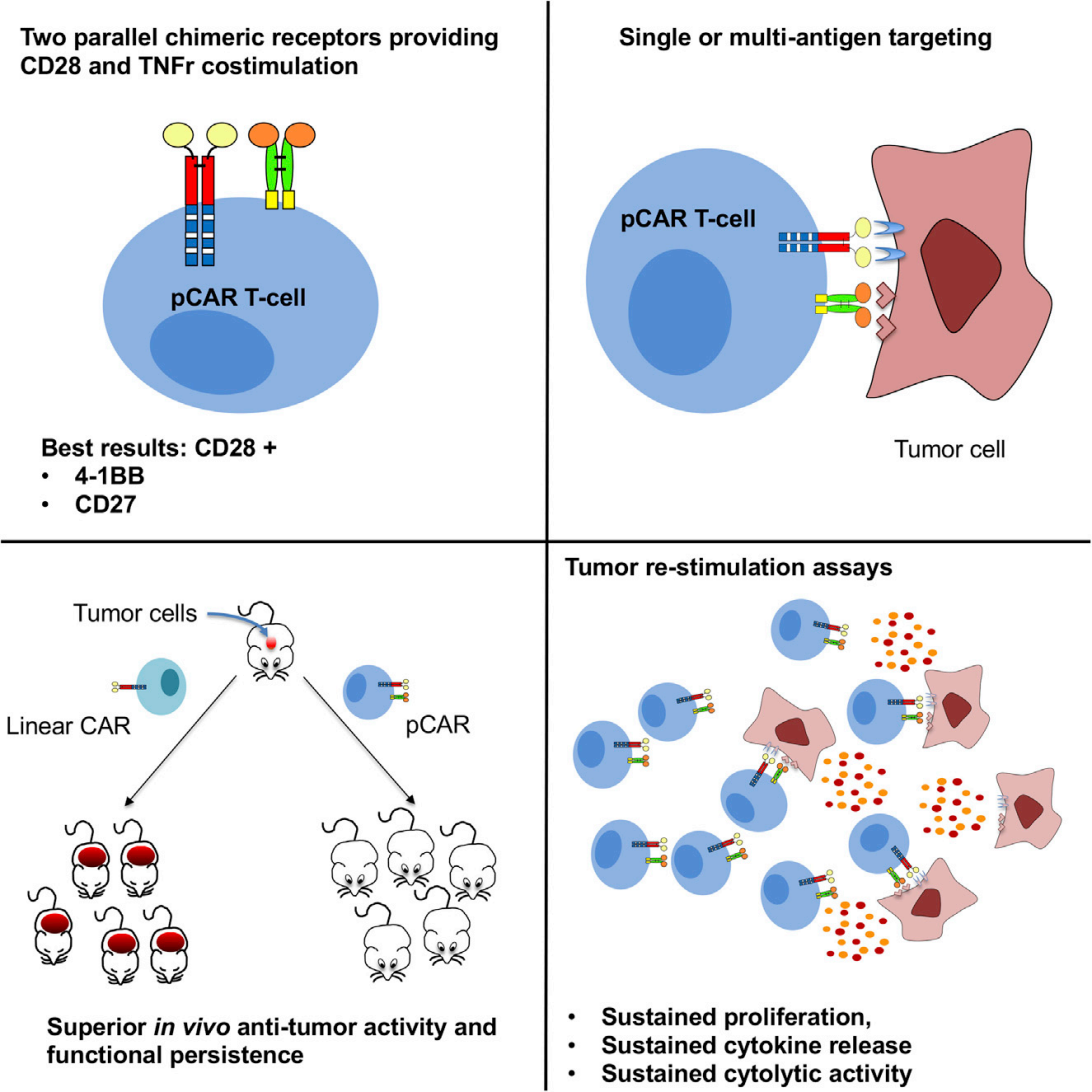
Expected outcomes

Expected outcomes are summarized in Figure 10. It is anticipated that pCAR T cells will enable the targeting of one or more antigens, leading to delivery of dual CD28 and TNFr co-stimulation. This results in sustained T cell proliferation, cytolytic activity, cytokine release, functional persistence *in vivo* (Muliaditan et al., 2021) and enhanced anti-tumor activity.

## Quantification and statistical analysis

Statistical analysis was performed using GraphPad Prism 9 software. Statistical comparison between two groups was undertaken using an unpaired Student *t*-test. Comparison of three or more groups was performed using one-way or two-way ANOVA with multiple comparisons, when one or two independent variables were present respectively. Survival was compared using a Log rank (Mantel-Cox) test. Homoscedasticity of residual variance and normality assumptions were met*.*

## Limitations

*In vivo* analysis of human CAR T cell functionality using immunocompromised mice is limited by many factors. Since NSG mice do not have human hematopoietic cells, it is difficult to predict how CAR T cells might overcome immunosuppressive cellular factors, particularly in the case of solid malignancies. Moreover, CAR T cells may not be exposed in an NSG model to the same immune suppressive checkpoint molecules, cytokines, and metabolic stresses which may be present in a naturally occurring tumor.

Using healthy donors as a source of PBMCs often results in successful transduction and expansion of CAR T cells. However, in a clinical setting when using autologous patient T cells, prior exposure to treatments such as chemotherapy and radiotherapy may compromise the quality and yield of CAR T cells.

## Troubleshooting

### Problem 1

Transduction of primary human CAR T cells can fail owing to poor quality transient viral vector (refer to corresponding protocol steps 15–19).

### Potential solution

High quality plasmids are required for efficient transfection of HEK293T cells. This can generally be achieved using commercially available kits and plasmid quality can be confirmed by measuring the ratio of absorbance at 260 nm and 280 nm. An A260/A280 ratio of above 1.8 generally indicates that plasmid quality is satisfactory. HEK293T cells should be of low passage number when transfected. Advanced passage number or failure to prevent over-confluence can significantly compromise viral titer. Finally, addition of an excess of activated T cells to RetroNectin® coated plasticware can compromise gene transfer efficiency.

### Problem 2

Low yields of pCAR T cells post expansion (refer to corresponding protocol step 43).

### Potential solution

A number of issues warrant consideration in this circumstance. Ensure that medium is fresh, in particular since L-glutamine undergoes degradation within about 2 weeks. While routine titration of retroviral vector is not usually necessary, in advertent addition of an excess number of viral particles can compromise T cell expansion. Furthermore, target antigens recognized by some pCARs may be found on activated T cells, leading to fratricide. Use of blocking antibodies directed against such targets can enable T cell expansion in this circumstance (Michaux et al., 2022).

### Problem 3

Undertaking functional studies using primary human CAR T cells can be unpredictable due to donor-to-donor variability (refer to corresponding protocol steps 61–79). T cell fitness and subsets within T cell populations can vary significantly between donors and manifest as inconsistent functionality of CAR T cells. Expansion, cytotoxicity, *in vivo* engraftment, persistence, and CD4/8 ratio can also be difficult to reproduce with consistency.

### Potential solution

The primary solution to this issue is to perform each experiment with multiple biological replicates.

### Problem 4

Tumor engraftment may fail in NSG mice following missed i.p. injection. Similarly, false negative BLI emission values may be obtained for the same reason (refer to corresponding protocol steps 70–79).

### Potential solution

Very few tumor cell lines will have 100% engraftment rates. Tumor cells left in PBS for extended periods of time before injection will greatly reduce engraftment rates. It may be useful to inject tumor in 3–4 additional mice to ensure that group sizes are sufficient for meaningful analysis. Tumor growth in PBS treated mice tends to be most homogeneous meaning that a smaller group size may be tolerated than is the case for T-cell-treated mice. If all animals engraft tumor successfully, it is recommended to add additional mice to the key T cell treatment groups.

Suspected failed i.p. injection of luciferin is generally readily identifiable because of the trend of BLI emission in affected mice. If repeat luciferin injection yields a positive reading, the spurious negative value should be omitted from data analysis.

### Problem 5

Inadequate anti-tumor activity of some pCAR configurations.

### Potential solution

Careful optimization of both receptors within a pCAR may be required. For example, with poorly accessible epitopes that lie close to the tumor cell membrane, there may be a need for an extended spacer domain (Guest et al., 2005) or flexible hinge (Wilkie et al., 2008). While the pCAR platform works over a range of affinities of CAR and CCR targeting moiety, excessive or inadequate affinity may also compromise function. Finally, distinct co-stimulatory domains may be required for optimal function in distinct immune subsets, such as CD4^+^ and CD8^+^ αβ T cells, γδ T cells and invariant NKT cells.

## Resource availability

### Lead contact

Further information and requests for resources and reagents should be directed to and will be fulfilled by the lead contact Dr John Maher (john.maher@kcl.ac.uk).

### Materials availability

Reagents generated in this study will be made available on request, but we may require a payment and/or a completed Materials Transfer Agreement if there is potential for commercial application.

## Data Availability

The datasets supporting this protocol, and constructs described or used in Figures, have not been deposited in a public repository but are available from the corresponding author upon request. There was no new code developed as part of this study**.**

## References

[bib1] Davies D.M., Foster J., van der Stegen S.J.C., Parente-Pereira A.C., Chiapero-Stanke L., Delinassios G.J., Burbridge S.E., Kao V., Liu Z., Bosshard-Carter L. (2012). Flexible targeting of ErbB dimers that drive tumorigenesis by using genetically engineered T cells. Mol. Med..

[bib2] Guest R.D., Hawkins R.E., Kirillova N., Cheadle E.J., Arnold J., O'Neill A., Irlam J., Chester K.A., Kemshead J.T., Shaw D.M. (2005). The role of extracellular spacer regions in the optimal design of chimeric immune receptors: evaluation of four different scFvs and antigens. J. Immunother..

[bib3] Michaux A., Mauen S., Breman E., Dheur M.S., Twyffels L., Saerens L., Jacques-Hespel C., Gauthy E., Agaugue S., Gilham D.E., Sotiropoulou P.A. (2022). Clinical grade manufacture of CYAD-101, a NKG2D-based, first in class, non-gene-edited allogeneic CAR T-cell therapy. J. Immunother..

[bib4] Muliaditan T., Halim L., Whilding L.M., Draper B., Achkova D.Y., Kausar F., Glover M., Bechman N., Arulappu A., Sanchez J. (2021). Synergistic T cell signaling by 41BB and CD28 is optimally achieved by membrane proximal positioning within parallel chimeric antigen receptors. Cell Rep. Med..

[bib5] Rivière I., Brose K., Mulligan R.C. (1995). Effects of retroviral vector design on expression of human adenosine deaminase in murine bone marrow transplant recipients engrafted with genetically modified cells. Proc. Natl. Acad. Sci. U S A.

[bib6] Stortelers C., van de Poll M.L.M., Lenferink A.E., Gadellaa M.M., van Zoelen C., van Zoelen E.J.J., van Zoelen E.J. (2002). Epidermal growth factor contains both positive and negative determinants for interaction with ErbB-2/ErbB-3 heterodimers. Biochemistry.

[bib7] Stortelers C., van der Woning S.P., Jacobs-Oomen S., Wingens M., van Zoelen E.J. (2003). Selective formation of ErbB-2/ErbB-3 heterodimers depends on the ErbB-3 affinity of epidermal growth factor-like ligands. J. Biol. Chem..

[bib8] Szymczak A.L., Workman C.J., Wang Y., Vignali K.M., Dilioglou S., Vanin E.F., Vignali D.A.A. (2004). Correction of multi-gene deficiency in vivo using a single 'self-cleaving' 2A peptide-based retroviral vector. Nat. Biotechnol..

[bib9] van Schalkwyk M.C., Papa S.E., Jeannon J.P., Urbano T.G., Spicer J.F., Maher J. (2013). Design of a phase I clinical trial to evaluate intratumoral delivery of ErbB-targeted chimeric antigen receptor T-cells in locally advanced or recurrent head and neck cancer. Hum. Gene Ther. Clin. Dev..

[bib10] van Schalkwyk M.C.I., van der Stegen S.J.C., Bosshard-Carter L., Graves H., Papa S., Parente-Pereira A.C., Farzaneh F., Fisher C.D., Hope A., Adami A., Maher J. (2021). Development and validation of a good manufacturing process for IL-4-driven expansion of chimeric cytokine receptor-expressing CAR T-cells. Cells.

[bib11] Vardhana S.A., Hwee M.A., Berisa M., Wells D.K., Yost K.E., King B., Smith M., Herrera P.S., Chang H.Y., Satpathy A.T. (2020). Impaired mitochondrial oxidative phosphorylation limits the self-renewal of T cells exposed to persistent antigen. Nat. Immunol..

[bib12] Wherry E.J., Kurachi M. (2015). Molecular and cellular insights into T cell exhaustion. Nat. Rev. Immunol..

[bib13] Whilding L.M., Halim L., Draper B., Parente-Pereira A.C., Zabinski T., Davies D.M., Maher J. (2019). CAR T-cells targeting the integrin αvβ6 and Co-expressing the chemokine receptor CXCR2 demonstrate enhanced homing and efficacy against several solid malignancies. Cancers.

[bib14] Whilding L.M., Parente-Pereira A.C., Zabinski T., Davies D.M., Petrovic R.M.G., Kao Y.V., Saxena S.A., Romain A., Costa-Guerra J.A., Violette S. (2017). Targeting of aberrant αvβ6 integrin expression in solid tumors using chimeric antigen receptor-engineered T cells. Mol. Ther..

[bib15] Wilkie S., Picco G., Foster J., Davies D.M., Julien S., Cooper L., Arif S., Mather S.J., Taylor-Papadimitriou J., Burchell J.M., Maher J. (2008). Retargeting of human T cells to tumor-associated MUC1: the evolution of a chimeric antigen receptor. J. Immunol..

[bib16] Xiong A.S., Yao Q.H., Peng R.H., Duan H., Li X., Fan H.Q., Cheng Z.M., Li Y. (2006). PCR-based accurate synthesis of long DNA sequences. Nat. Protoc..

